# Caesarean section rate and postnatal bed occupancy: a retrospective study replacing assumptions with evidence

**DOI:** 10.1186/s12913-018-3570-3

**Published:** 2018-10-05

**Authors:** Subhadeep Roy, L Montgomery Irvine

**Affiliations:** 1 Department of Obstetrics & Gynaecology, Watford General Hospital, Watford, WD18 0HB UK; 2 Department of Obstetrics & Gynaecology, Cambridge University Hospitals NHS Foundation Trust, Hills Road, Cambridge, CB2 0QQ UK

**Keywords:** Caesarean section, Postnatal bed occupancy, Post-caesarean length of stay, Caesarean on request, Patient choice

## Abstract

**Background:**

Obstetric units across the UK face resource pressures alongside a rising rate of Caesarean section (CS). It is assumed that this places a further burden in the form of postnatal bed demands. The number of inpatient beds has fallen nationally, and this may be used to justify attempts to restrict the CS rate. We set out to replace such assumptions with evidence. We did not find any similar contemporary analysis in a literature search.

**Methods:**

The postnatal length of stay (LOS) of women delivering at Watford General Hospital, a large unit hosting around 5500 deliveries per annum, was stratified by mode of delivery. Differences within and across time periods were analysed.

**Results:**

The CS rate rose from 14.5% in 1995 to 30.9% in 2015. The mean LOS post-CS declined from 4.2 to 2.4 days. These data were statistically significant to *p* < 0.001. Over this period the standardised total postnatal bed use for all delivery modes fell from 11083 days to 7894 days. A 113% rise in the CS rate was accommodated by only a 19.8% rise in postnatal bed use attributable to CS patients.

**Conclusions:**

Whatever pressures may be exacerbated by the rising CS rate, bed occupancy is not one of them. In discussion we widen our argument to suggest that resource pressures should not be used to justify limitations in the CS rate.

**Electronic supplementary material:**

The online version of this article (10.1186/s12913-018-3570-3) contains supplementary material, which is available to authorized users.

## Background

The NHS, the world’s fifth largest employer [1], is under fiscal strain. For some time now funding has not kept pace with demand and maternity services, accounting for approximately £2.5 billion out of the £101 billion budget in England [2], have been no exception. It remains proudly a quite unique healthcare system, maintaining the model of service based on clinical need, free at the point of care.

Though there exist regional variations, the rate of CS globally [3, 4] and within NHS maternity units [5] continues to rise. The reasons behind this are many and include an expanding cohort of older mothers with medical co-morbidities [6] and raised body mass index [7]. It is also likely that changing risk perceptions on the part of both pregnant women and clinicians delivering obstetric care play a part. It may be thought to follow that this trend places a heavy burden upon maternity unit resources. Supporting this assumption it was estimated that the average cost to the NHS in 2015 of a CS delivery was £3371 compared with £1514 for normal vaginal deliveries [8]. However the cost of an emergency CS at £3820 was significantly higher than that of an elective CS at £2922 and cost forecasting for planned vaginal delivery must take this into account. In the UK elective versus emergency CS are defined by guidance from the Royal College of Obstetricians & Gynaecologists [9]. Elective CS includes only those cases where there is no maternal or foetal compromise and delivery is scheduled as per the convenience of the woman and the clinical team. All other CS deliveries are classified as emergency procedures.

The 2011 NICE guideline [10] regarding CS concluded that elective maternal request CS: ‘could be considered a cost effective alternative to planned vaginal birth’. Much data regarding the risks, benefits and resource implications of CS delivery suffers from a failure to clearly separate the entities of elective and emergency CS.

Another factor to consider is the implication on fixed infrastructure. In this study we focus upon the impact of rising rates of CS (both elective and emergency) upon bed occupancy in our maternity unit. It is a fact that average length of postnatal stay is greatest after CS delivery, and it is true that the tenure of successive health secretaries has seen a decline in the inpatient bed count. This reduction has not spared maternity units, which have lost around two thousand beds across England between 1998 and 2009 [11]. Therefore it may be thought surprising that these two realities have coexisted thus far with little tension.

How, then, has an increasing CS rate impacted upon postnatal bed use in our busy maternity unit? It is important to stress that our analysis sets out only to answer this specific question in the context of our department. We make no attempt to explain the rising CS rate that is demonstrated, which is quite a separate matter.

## Methods

This study was conducted at Watford General Hospital, part of the West Hertfordshire Hospitals NHS Trust (where the authors work). It is a large hospital providing acute services to a population of around 500 000 people. The maternity service provides comprehensive care for approximately 5500 women per year, making it one of the busiest units in the East of England region. It serves a diverse population with no obvious demographic features that would place it out of line with other units in urban locations. We are unaware of any specific demographic changes that have occurred over the period covered by the dataset but cannot exclude this.

Retrospective data for the years 1995 to 2015 were obtained to determine the proportion of deliveries by CS each year, and the postnatal LOS stratified by mode of delivery. The study population consisted of all women recorded in the local birth register as having delivered during that period. Data has been entered since 1988 on the Ciconia Maternity information System (CMiS) supplied by HD Medical Group. A wide range of data is entered for audit purposes and is made available to the Department of Health and NHS England whenever required for performance analysis. Data entry is performed by midwives and clerical staff at the point of discharge. CS rate data are accurate since the point of inception of CMiS but we noted shortcomings in LOS data in the earlier years. It is for this reason that the period 1995 to 2015 was selected and within this range there were two individual years, 2011 and 2012, when the LOS of a significant proportion of patients was not entered. These years were therefore excluded from figures and calculations involving bed days used. The reason for this aberration remained unclear following internal consultation.

Data were analysed in raw form as far as the direct CS rate and average LOS for different modes of delivery are concerned. To enable relevant comparison of the postnatal bed use from year to year a further dataset was derived from the raw data to control for the variable of total number of deliveries per year, which is subject to natural fluctuation.

We were fortunate in this study to deal with robust data and large sample sizes. A two sample T-test between percents was used to verify the significance of the changing CS rate and the χ-squared test was applied to the difference in LOS post-CS between 1995 and 2015. A *p* value of less than 0.05 was chosen as an appropriate level of significance prior to analysis.

## Results

**Fig. 1:**
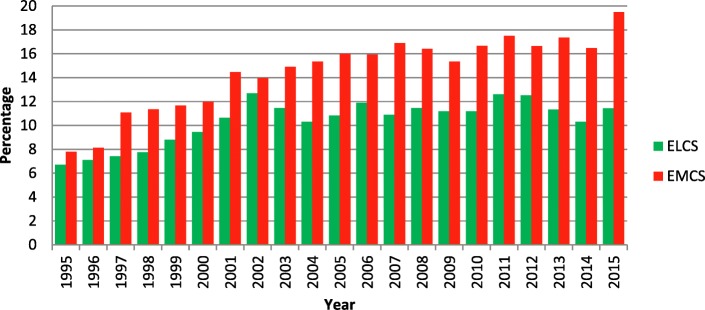
The CS rate at Watford General Hospital as a percentage of all deliveries has risen from 14.5% in 1995 to 30.9% in 2015. It can be seen that the emergency CS rate has risen more than the elective rate. In 1995 53.8% of the 865 CS deliveries were performed as an emergency and in 2015 the corresponding figure was 63.1% of the 1631 CS deliveries

Between 1995 and 2015 the CS rate rose from 14.5 to 30.9% of all deliveries. This is a statistically significant difference with *p* < 0.001. The emergency CS rate expressed as a proportion of all CS cases has risen from 53.8 to 63.1% over the same period. These trends are illustrated in Fig. 1.

**Fig. 2:**
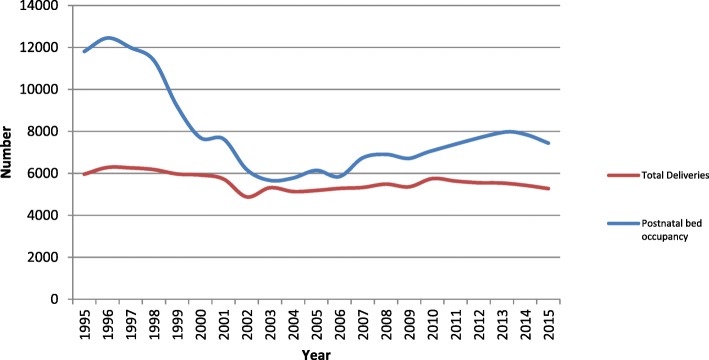
This raw non-standardised data captures a snapshot of the maternity unit activity at Watford General Hospital between 1995 and 2015. Bed days used post-delivery has fallen significantly from 11810 days in 1995 to 7442 days in 2015 despite a relatively stable number of deliveries through the years

Figure 2 displays the raw data concerning total number of deliveries and total number of postnatal bed days used in each year of the study period. A total of 5960 deliveries in 1995 required 11810 postnatal bed days while only 7442 bed days were needed in 2015 to accommodate 5273 deliveries. When standardised to control for the number of deliveries per year, postnatal bed use for all deliveries has fallen from 11083 days in 1995 to 7894 days in 2015.

**Fig. 3:**
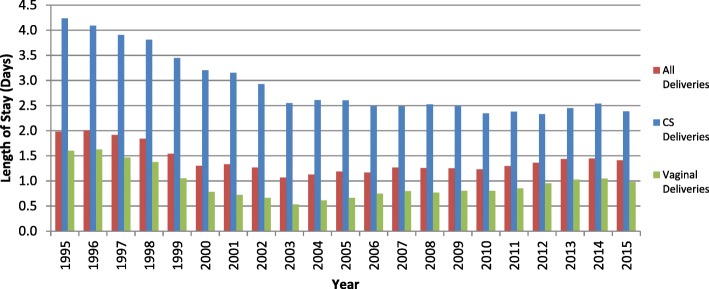
This clearly demonstrates a trend towards falling LOS that is most notable for CS deliveries with average LOS for this group falling from 4.2 days in 1995 to 2.4 days in 2015. For all patients regardless of mode of delivery the LOS fell from 2.0 days to 1.4 days and excluding CS patients the LOS fell from 1.6 to 1.0 days over the same period

**Fig. 4:**
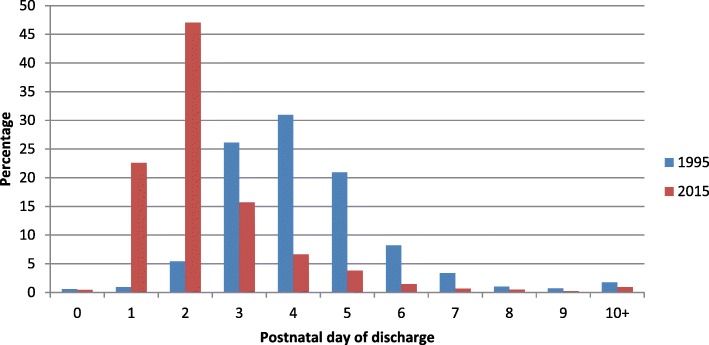
This directly compares the postnatal day of discharge for CS deliveries in 1995 and 2015. A clear distributional change is seen with the modal day of discharge falling from day 4 to day 2

The basis for this trend in reduced postnatal bed days used is shown in Fig. 3 which shows that the average LOS for CS patients fell from 4.2 days in 1995 to 2.4 days in 2015. This was statistically significant change with *p* < 0.001. Illustrations for vaginal deliveries and all deliveries are also shown. Figure 4 concerns only the LOS for CS deliveries and illustrates the distributional change in day of discharge between 1995 and 2015 with the modal day of discharge subsiding from day 4 to day 2. This illustration is widened to demonstrate the evolution of LOS for all modes of delivery in Additional file 1: Figure S1 and Additional file 2: Figure S2.

**Fig. 5:**
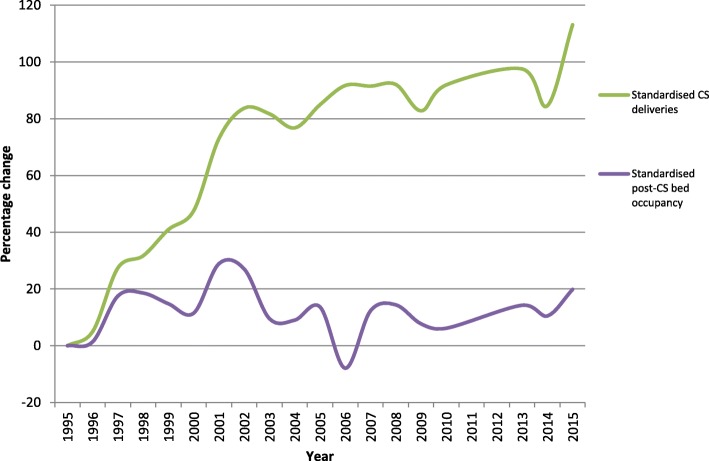
This compares the percentage change in the CS rate with the percentage change in postnatal bed use attributable to CS. Both parameters are calculated for each year in succession with reference to the 1995 figure. The data have been standardised to control for the raw number of deliveries per year. At the end of the twenty-year period a 113% rise in the CS rate was accommodated by only a 19.8% rise in bed use post-CS. The underlying changes are displayed in Additional file 3: Figure S3 and Additional file 4: Figure S4

Figure 5 illustrates how over the 20-year period a rise in the CS rate of 113% has been accommodated by only a 19.8% rise in postnatal bed use attributable to CS patients. Additional file 3: Figure S3 and Additional file 4: Figure S4 demonstrate the underlying changes that account for this using standardised data. The 14.5% CS rate in 1995 required 11083 bed days for all postnatal patients of which 4248 bed days were used for CS patients, while the 30.9% CS rate in 2015 was accommodated by 7894 bed days for all postnatal patients of which 5848 bed days were used for CS patients.

## Discussion

In this study we found that the rate of CS at our unit has risen from 14.5 to 30.9% between 1995 and 2015 which is broadly comparable to national figures. This is due to a variety of factors most importantly changing risk perceptions and clinician behaviour. Some of the medical reasons for the change have previously been considered by the senior author in the context of this unit [12–14].

While there are of course important clinical correlations to be made of the rising CS rate in terms of maternal and neonatal health, particularly with reference to the increased incidence of abnormalities of placentation with a rising CS rate [15], it is also important to consider non-clinical consequences such as resource provision and utilisation. We discovered that contrary to expectation the post-CS LOS has fallen by so great an extent over the study period that the rising CS rate has allowed for a decline in postnatal bed usage. We calculated that had the average LOS post-CS remained the same in 2015 as it was in 1995 an extra 3000 postnatal bed days would have been required. It is estimated that an extra postnatal bed day costs £402 [8] meaning the total additional cost to our Trust would have been over £1.2 million. Using the available data it is not possible to directly comment on how this compares with increased costs associated with the procedure itself. We are not aware of any change in policy regarding timing of discharge post-Caesarean locally or within the wider NHS that would specifically account for the fall in length of stay.

It is crucial that elective and emergency CS are not considered as a single entity. They are very different procedures with distinct risk-benefit profiles. Likewise the resource implications also differ. We were unable to further subdivide our LOS data by category of CS as the database does not provide this breakdown. Further studies should look specifically at the LOS data for emergency versus elective CS and our hypothesis is that the elective group would require a significantly shorter postoperative stay. It is disappointing to note that the rise in our emergency CS rate from 7.8 to 19.5% has greatly outpaced the rise in our elective CS rate from 6.7 to 11.4% as the former is both costlier and carries greater risks for mother and baby.

In day to day practice a difficulty faced by obstetricians is the dilemma of CS on maternal request. The NICE guideline is clear that if there is no obvious contraindication to CS and the woman wishes for a CS following detailed counselling regarding the risks and benefits, it should be offered [10]. This guideline is not uniformly applied in practice and many units have their own policies in place. At one end of this spectrum are units with a blanket policy to deny CS on request. This can sometimes lead to disappointment [16] on the part of women, some of whom having fully understood the pros and cons of a trial of labour versus elective CS would prefer to opt for the risk profile of elective CS rather than tolerate any possibility of foetal intrapartum distress, or emergency CS and instrumental delivery - the rate of the latter two together equalling nearly 30% in normal labour [17]. Such patients may be more likely to litigate if there are adverse outcomes, and they may also rebook with an alternative unit late in pregnancy which is an unsatisfactory outcome for all parties.

The justification units offer regarding policies against maternal request CS may include that they feel a diversion of resources towards elective CS without obstetric indication may be to the detriment of those women labouring normally. The possibility of postnatal bed blocking is one such concern. In a healthcare infrastructure starved of investment this would be unfair. We believe our data reveals the possibility that a more relaxed policy on maternal request CS, the demand for which is not clearly demonstrated to be significant [18], may not impact adversely upon the functioning of a maternity unit. Perceived lack of choice is an issue in maternity care [19] and the avoidance of even a small number of patients who feel they have not been offered the options promised to them by NICE and advertised boldly by NHS Choices [20] is perhaps worth a trial of such a policy. Ours is an important pilot study that strongly refutes the commonly held belief that the rising CS rate has placed greater strain on inpatient maternity beds. It would be beneficial to aid strategic planning for postnatal capacity in future to extend this study to a wider geographical range and rigorously examine the difference between elective and emergency CS. It was highlighted that the rise in our emergency CS rate has outpaced the elective CS rate. It is likely that this is primarily due to a reduced threshold to intervene based on changing risk perceptions of both patient and clinician, but it is important to consider any means by which we could better predict in the antenatal period which women are likely to require emergency CS in labour and attempt to forestall this by offering them elective CS which is safer and more cost-effective.

In conjunction with NICE data regarding the limited cost differential between elective CS and trial of labour such wider verification of our findings would lend weight to an argument against the banning of CS on maternal request on the grounds of judicious use of resources. If we are to be a modern forward-looking specialty it is paramount that we ensure decisions around mode of delivery are made solely with safety and patient choice in mind.

## Conclusion

Our analysis demonstrates that although the rate of CS has increased dramatically between 1995 and 2015, due to the marked decline in the average length of stay post-CS no additional demands have been placed upon the postnatal inpatient capacity at Watford General Hospital. We suggest that arguments proposing a targeted reduction in the CS rate or advocating against further rises in the CS rate should not be based on cost and resource issues and should be guided entirely by patient safety and patient choice. Further work is required to differentiate outcomes, bed pressures and costs arising from emergency as opposed to elective CS.

## Additional files

Additional file 1: **Figure S1.** This is a snapshot of postnatal LOS in 1995 subdivided by method of delivery. The modal day of discharge for CS deliveries was postnatal day 4 and the modal day of discharge for vaginal deliveries was postnatal day 1. (DOCX 23 kb)

Additional file 2: **Figure S2.** This is a snapshot of postnatal LOS in 2015 subdivided by method of delivery. The modal day of discharge for CS deliveries was postnatal day 2 and the modal day of discharge for vaginal deliveries was postnatal day 0 (the day of delivery). (DOCX 23 kb)

Additional file 3: **Figure S3.** This demonstrates a trend towards reduced overall postnatal bed use for all modes of delivery (adjusted to control for the raw number of deliveries each year) in the face of a rising CS rate. The CS rate in 1995 was 14.5% and the total postnatal bed use was 11083 days. The corresponding figures for 2015 were 30.9% and 7894 days respectively. (DOCX 23 kb)

Additional file 4: **Figure S4.** This demonstrates a very shallow rise in the postnatal bed use accounted for by CS deliveries (adjusted to control for the raw number of deliveries each year) in the face of a rising CS rate. The CS rate in 1995 was 14.5% and the postnatal bed use attributable to that was 4248 days. The CS rate in 2015 was 30.9% but the postnatal bed use attributable to that has only risen to 5848 days. (DOCX 23 kb)

## Abbreviations

CMiS: Ciconia Maternity information Systems
CS: Caesarean section
LOS: length of stay
