# Supplementary material for: Caesarean section rate and postnatal bed occupancy: a retrospective study replacing assumptions with evidence
Source: BMC Health Serv Res. 2018 Oct 5;18:760. doi: 10.1186/s12913-018-3570-3 (PMC6173897; doi:10.1186/s12913-018-3570-3)
Supplement: Supplementary file 2 [file 12913_2018_3570_MOESM2_ESM.docx]

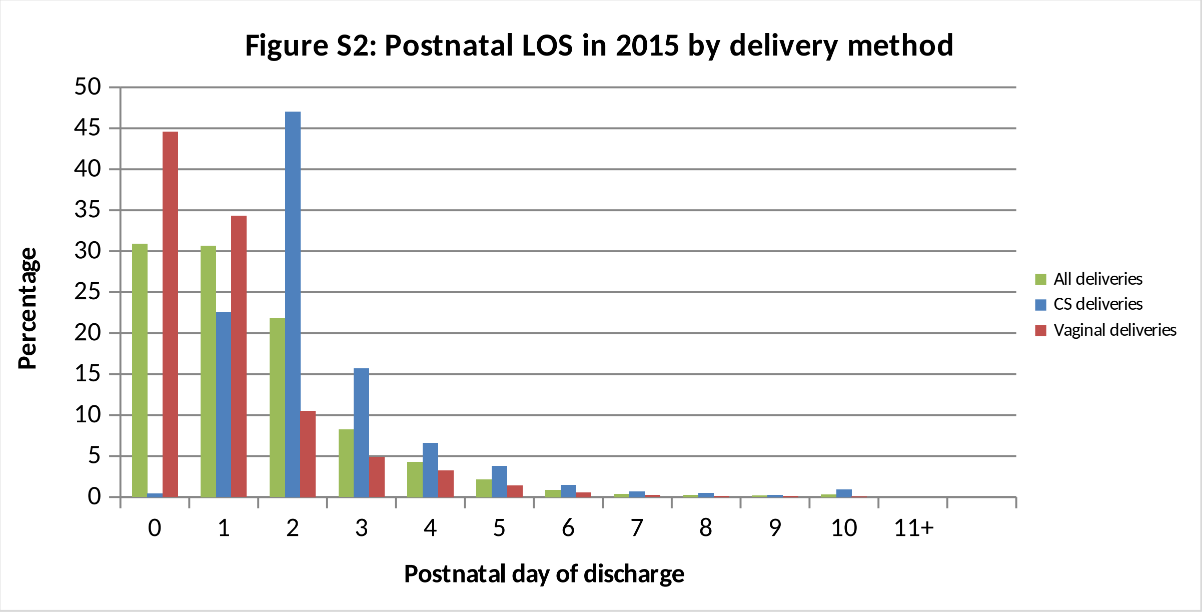
Figure S2: Postnatal LOS in 2015 by delivery method
Percentage
50
45
40
35
30
25
20
15
10
5
0
0
1
2
3
4
5
6
7
8
9
10
11+
Postnatal day of discharge
All deliveries
CS deliveries
Vaginal deliveries
