# Supplementary material for: Caesarean section rate and postnatal bed occupancy: a retrospective study replacing assumptions with evidence
Source: BMC Health Serv Res. 2018 Oct 5;18:760. doi: 10.1186/s12913-018-3570-3 (PMC6173897; doi:10.1186/s12913-018-3570-3)
Supplement: Supplementary file 4 [file 12913_2018_3570_MOESM4_ESM.docx]

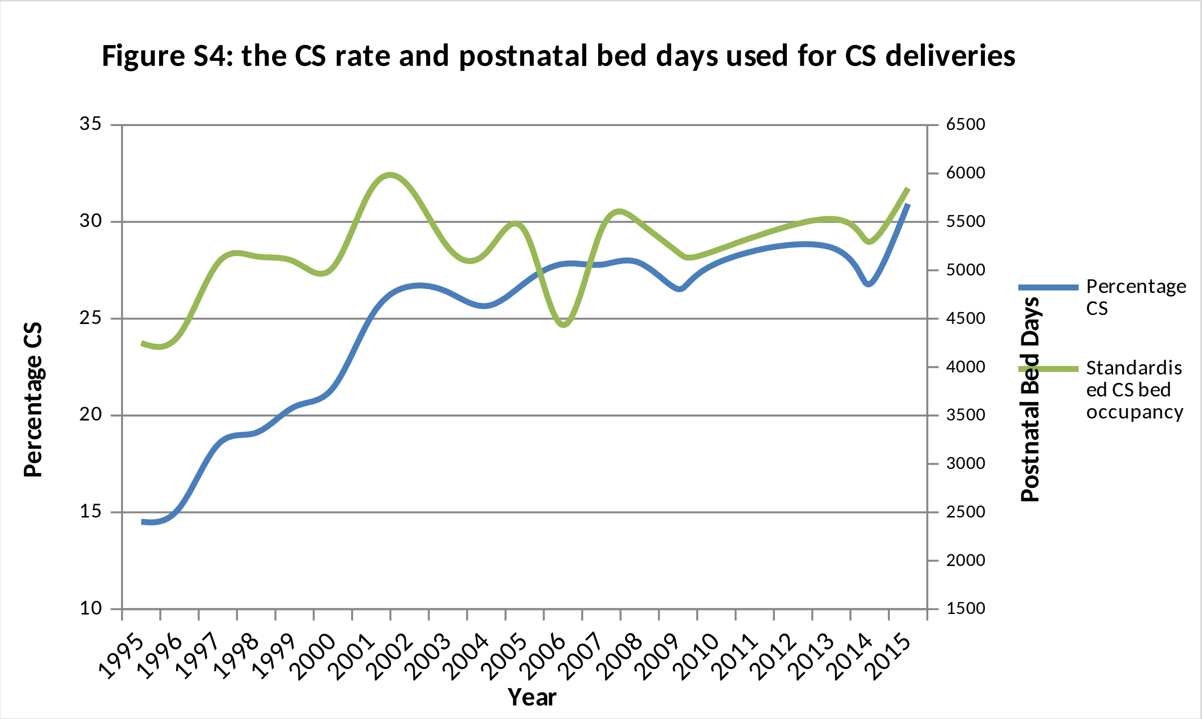
Figure S4: the CS rate and postnatal bed days used for CS deliveries
Percentage CS
35
30
25
20
15
10
Postnatal Bed Days
6500
6000
5500
5000
4500
4000
3500
3000
2500
2000
1500
1995
1996
1997
1998
1999
2000
2001
2002
2003
2004
2005
2006
2007
2008
2009
2010
2011
2012
2013
2014
2015
Year
Percentage CS
Standardised CS bed occupancy
